# Maturation of Paracetamol Elimination Routes in Preterm Neonates Born Below 32 Weeks of Gestation

**DOI:** 10.1007/s11095-023-03580-3

**Published:** 2023-08-21

**Authors:** Yunjiao Wu, Swantje Völler, Elke H. J. Krekels, Daniëlla W. E. Roofthooft, Sinno H. P. Simons, Dick Tibboel, Robert B. Flint, Catherijne A. J. Knibbe

**Affiliations:** 1 Division of Systems Pharmacology and Pharmacy, Leiden Academic Centre for Drug Research, Leiden University, Leiden, The Netherlands; 2 Department of Pediatrics, Division of Neonatology, Erasmus MC Sophia Children’s Hospital, Rotterdam, The Netherlands; 3 Department of Pediatric Surgery, Erasmus University MC–Sophia Children’s Hospital, Rotterdam, The Netherlands; 4 Department of Hospital Pharmacy, Erasmus University Medical Center, Rotterdam, The Netherlands; 5 Department of Clinical Pharmacy, St Antonius Hospital, PO Box 2500, 3430 EM Nieuwegein, The Netherlands

**Keywords:** glucuronidation, metabolism, oxidation, pharmacokinetics, sulfation

## Abstract

**Purpose:**

Despite being off-label, intravenous paracetamol (PCM) is increasingly used to control mild-to-moderate pain in preterm neonates. Here we aim to quantify the maturation of paracetamol elimination pathways in preterm neonates born below 32 weeks of gestation.

**Methods:**

Datasets after single dose (rich data) or multiple doses (sparse data) of intravenous PCM dose (median (range)) 9 (3–25) mg/kg were pooled, containing 534 plasma and 44 urine samples of PCM and metabolites (PCM–glucuronide, PCM–sulfate, PCM–cysteine, and PCM–mercapturate) from 143 preterm neonates (gestational age 27.7 (24.0–31.9) weeks, birthweight 985 (462–1,925) g, postnatal age (PNA) 5 (0–30) days, current weight 1,012 (462–1,959) g. Population pharmacokinetic analysis was performed using NONMEM® 7.4.

**Results:**

For a typical preterm neonate (birthweight 985 g; PNA 5 days), PCM clearance was 0.137 L/h, with glucuronidation, sulfation, oxidation and unchanged renal clearance accounting for 5.3%, 73.7%, 16.3% and 4.6%, respectively. Maturational changes in total PCM clearance and its elimination pathways were best described by birthweight and PNA. Between 500–1,500 g birthweight, total PCM clearance increases by 169%, with glucuronidation, sulfation and oxidation clearance increasing by 347%, 164% and 164%. From 1–30 days PNA for 985 g birthweight neonate, total PCM clearance increases by 167%, with clearance via glucuronidation and oxidation increasing by 551%, and sulfation by 69%.

**Conclusion:**

Birthweight and PNA are the most important predictors for maturational changes in paracetamol clearance and its glucuronidation, sulfation and oxidation. As a result, dosing based on bodyweight alone will not lead to consistent paracetamol concentrations among preterm neonates.

**Supplementary Information:**

The online version contains supplementary material available at 10.1007/s11095-023-03580-3.

## Introduction

Despite being off-label, intravenous paracetamol (PCM) is increasingly used to control mild-to-moderate pain in preterm neonates and to spare the use of opioids in severe pain [1, 2]. Understanding the maturation in the different elimination pathways of PCM to subsequently describe the exposure of PCM and its metabolites across the heterogeneous population of preterm neonates is a necessary step towards its safe and effective use in this vulnerable population.

The metabolism of PCM mainly includes glucuronidation, sulfation and oxidation. In adults, about 55% and 30% of PCM is metabolized into glucuronide and sulphate conjugates, respectively. About 8–10% of an administered dose undergoes metabolism via the oxidative route, resulting in the formation of the toxic metabolite N-acetyl-p-benzoquinone imine (NAPQI). This metabolite is immediately conjugated with reduced glutathione, ultimately generating PCM-mercapturate and PCM-cysteine. Only 2–5% of administered PCM is excreted unchanged in the urine [3, 4]. Throughout childhood, the contribution of each metabolic pathway changes considerably, with sulfation being the dominant pathway in neonates and the contribution of glucuronidation progressively increasing [5, 6].

PCM pharmacokinetics have been studied in the neonatal population albeit with limited focus on the contribution of the different pathways to overall clearance of PCM [7–14]. Regarding metabolism of PCM in neonates, only a few neonatal population pharmacokinetic (PopPK) studies described the maturation of glucuronidation and sulfation and even fewer the oxidation pathway [7, 9], especially in the most preterm neonates who require prolonged stay in the neonatal intensive care units and suffer from incomplete hepatic and renal maturation.

The aim of this study was to quantify the maturation of PCM glucuronidation, sulfation and oxidation as part of total PCM clearance across a population of extremely to very preterm neonates (gestational age < 32 weeks) with varying postnatal age (PNA) after single or multiple intravenous PCM doses. For this, a PopPK model was developed based on measured PCM and metabolites in plasma and urine.

## Methods

### Study Population

Two datasets were pooled in this study. The first dataset was obtained from a previously published study [15] and consisted of data from 60 preterm neonates born before 32 weeks of gestation, who were included between October 2010 and October 2013 at the level three Neonatal Intensive Care Units of the Erasmus Medical Center—Sophia Children’s Hospital in Rotterdam and Isala Clinics in Zwolle, the Netherlands. Approval of the Ethics Review Committees from both hospitals and written informed consent from parents/legal guardians were obtained before study initiation (MEC-2009–250, National Trial Register 2290). The second dataset consisted of data from 83 neonates born before 32 weeks of gestation who were included in the Drug dosage Improvement in Neonates (DINO) study between September 2014 and July 2017. This study was conducted in four Dutch Neonatal Intensive Care Units. The Erasmus Medical Center ethics review board approved the protocols, and written informed consent from parents/legal guardians was obtained before study initiation (NL47409.078.14, MEC-2014–067, NCT02421068).

The detailed characteristics of patients in each dataset and the combined dataset are summarized in Table I.

**Table I:** Characteristics of Patients Included in the Analysis

	Dataset 1 Flint *et al*. (n = 60) [15]	Dataset 2 DINO-study (n = 83)	Combined dataset (n = 143)
Birthweight (g)	948 (462–1,550)	1,097 (540– 1,925)	985 (462–1,925)
Gestational age (week)	27.7 (24.0–31.1)	27.8 (24.4 – 31.9)	27.7 (24.0–31.9)
Postnatal age at start of treatment (days)	5 (0–10)	7 (0 – 30)	5 (0–30)
Postmenstrual age (weeks)	28.4 (24.9–31.7)	28.8 (24.7–35.1)	28.6 (24.7–35.1)
Current weight (g)	948 (462–1,550)	1,147 (560 – 1,959)	1,012 (462–1,959)
Small for gestational age, n (%)	32 (53.0)	18 (21.6)	50 (35.0)
Sex, n (%)			
Boy	28 (46.7)	47 (56.6)	75 (52.4)
Girl	32 (53.3)	36 (43.4)	68 (47.6)
Number of doses per individual, n (IQR)	1 (1–1)	10 (5–15)	2(1–11)
Duration of treatment (hours) (IQR)	Single dose	58 (28.8–139)	-
Dosage (mg/kg) (IQR)	10, 15, or 20	Loading dose: 19.0 (13.2–20.3) Maintenance: 9.5 (6.1–10.1)	9.7 (6.2–10.2)
Daily dosage (mg/kg/day) (IQR)	10, 15, or 20	29.8 (20.2–39.8)	29.1 (20.0–39.6)
Plasma samples, n	247	287	534
Plasma samples per individual, n	4 (3–5)	3 (1–13)	4 (1–13)
Number of individuals with urinary sample, n	44	0	44

All values are indicated as median (range) unless stated otherwise. IQR, interquartile range

### Dosing and Sampling Schedule

In dataset 1, 60 patients were randomized to receive one dose of PCM of 10, 15 or 20 mg/kg intravenously. PCM was administered via a 15-min infusion, before peripheral central venous catheter placement. Thereafter, blood samples were scheduled to be collected at different time points (20, 60, 240, 540 min or 15, 30, 120, 360, 720 min after start of the infusion). Urine samples were scheduled to be collected from the diaper over a continuous period of maximum 12 h after PCM administration. The actual urine collection interval and volume varied and were recorded. Plasma samples of all patients were available. Urine samples were successfully obtained from 44 patients, with each patient contributing one urine sample. PCM and metabolite concentrations were determined in both plasma and urine samples.

In dataset 2, the dosing schedule was based on current weight according to Wang *et al*. [13]. The dosing regimen could be adapted in individual patients by the treating physician. As a result, a loading dose (median 19.0 mg/kg, IQR 13.2–20.3) was given to 28 out of 83 subjects, and a median of 9.5 mg/kg (IQR 6.0–10.1) maintenance dose were given. Each patient received a median of 10 (IQR 5–14) maintenance doses with a median dosing interval of 6.1 h (IQR 5.7–7.4 h). Plasma samples were either scavenged from routine care or strategically drawn to determine PCM and metabolite concentrations. No urine samples were collected.

The observed plasma concentrations *versus* time after last dose (TAD) profiles for each dataset and compound are provided in Supplementary Fig. S1.

### Analytical Method

In dataset 1, plasma and urine concentrations of PCM, PCM–glucuronide, PCM–sulfate, PCM–cysteine, and PCM–mercapturate, were measured using high-performance liquid chromatography–electrospray ionization–tandem mass spectrometry (HPLC–MS/MS) at the Center for Human Toxicology, University of Utah (Salt Lake City, UT) [16]. For the plasma samples, the lower limits of quantification (LLOQ) were 0.05, 0.05, 0.05, 0.01 and 0.01 mg/L, and the percentages of samples below the lower limit of quantification (BLOQ) were 0%, 4.9%, 0.4%, 0% and 8.2% for PCM, PCM-glucuronide, PCM-sulfate, PCM-cysteine and PCM-mercapturate, respectively. For those BLOQ samples, the values obtained with the assay were still reported and these were used in the PopPK analysis. For the urine samples, the LLOQ were 0.2, 1.0, 1.0, 0.1 and 0.1 mg/L for the aforementioned compounds, respectively. None of the available samples were BLOQ.

In dataset 2, plasma concentrations of PCM, PCM–glucuronide, PCM–sulfate, PCM–cysteine, and PCM–mercapturate were measured by ultra-performance liquid chromatography-electrospray ionization-tandem mass spectrometry (UPLC-MS/MS) in laboratory of the Erasmus Medical Center Rotterdam [17]. The LLOQ was 0.05, 0.05, 0.09, 0.036 and 0.05 mg/L and the percentage of BLOQ samples were 3.4%, 4.1%, 0%, 0.3% and 2.8% for PCM, PCM-glucuronide, PCM-sulfate, PCM-cysteine and PCM-mercapturate, respectively. BLOQ samples with reported values were only partly available. When not available (n = 13), samples were not included in the analysis.

### Population Pharmacokinetics Model Development

A population pharmacokinetic model was developed using NONMEM V7.4.3 (ICON Development Solutions, Ellicott City, MD, USA), supported by Perl-speaks-NONMEM (PsN) 4.9.0, and interfaced by Pirana 2.9.9 (Certara USA, Inc., Princeton, NJ, USA). Processing and visualization of output from NONMEM were performed in R 4.2.3 (CRAN.R-project.org). Parameters were estimated using the first-order conditional estimation with interaction (FOCE + I) method. Differential equations were solved by use of the ADVAN6 subroutine (TOL = 6). Parameter precision was obtained with the R covariance matrix.

PCM-glucuronide, PCM-sulphate, PCM-mercapturate and PCM-cysteine were converted to equivalent PCM concentrations using the molecular weight of each compound (151.16, 327.29, 231.23, 312.24 and 270.30 g/mol for PCM, PCM-glucuronide, PCM-sulfate, PCM-mercapturate and PCM-cysteine, respectively). PCM-mercapturate and PCM-cysteine concentrations were summed up to represent the metabolites formed in the oxidation pathway. Urine concentrations and volumes of PCM and metabolites were included in NONMEM input dataset so that NONMEM could scale appropriately to urinary amounts. The concentrations of paracetamol and all metabolites were logarithmically transformed.

First, PCM concentrations in plasma were modeled, then the metabolite data in plasma and urine were added and analyzed simultaneously. For the structural models of PCM and metabolites, both one and two -compartment models were tested. The distribution volume of PCM-glucuronide, PCM-sulfate and PCM-oxidative pathway metabolites were set to a fraction of the central distribution volume of PCM.

To quantify the formation clearance of each metabolite, the fraction of total PCM CL going via each pathway was estimated using a parameterization that constrains all fractions between 0 and 1:

1 $${R}_{FT}={R}_{FG}+{R}_{FS}+{R}_{FO}+1$$

2 $$FG={R}_{FG}/{R}_{FT}$$

3 $$FS={R}_{FS}/{R}_{FT}$$

4 $$FOX={R}_{FO}/{R}_{FT}$$

5 $$FR=1/{R}_{FT}$$

where *R*_*FG*_, *R*_*FS*_ and *R*_*FO*_ are the fold difference of the glucuronidation, sulfation and oxidation pathway CL, respectively, relative to the unchanged renal excretion CL of PCM. Then the exact fraction of glucuronidation (*FG* in Eq. 2), sulfation (*FS* in Eq. 3), oxidation (*FOX* in Eq. 4), and renal excretion (*FR* in Eq. 5) were calculated by diving their respective fold by *R*_*FT*_, which represents how many times unchanged renal PCM excretion CL equals the total PCM CL.

The stochastic model included interindividual variability (IIV) and residual variability (RUV). The IIV for each parameter was implemented assuming a log-normal distribution:

6 $$P_i=P_p\times e^{\eta_i};\;\eta_i\sim N(0,\omega^2)$$

where ${P}_{i}$ is the individual parameter estimate for the *i*th individual, ${P}_{p}$ is the population estimate for parameter *P*, and ${\eta }_{i}$ is a random variable for the *i*th individual from a normal distribution with a mean of zero and an estimated variance of *ω*^2^.

For RUV, a combined proportional and additive residual error model in the log-domain was used:

7 $$\begin{array}{c}\log\left(C_{ij}\right)=Log\left(C_{{ipred}_{ij}}\right)+{SD\times\varepsilon}_{ij};\;\varepsilon_{ij}\sim N(0,1^2)\\SD=\sqrt{{SD}_1^2+\left(\frac{{SD}_2}{C_{{ipred}_{ij}}}\right)^2}\end{array}$$

where ${C}_{ij}$ is the observed concentration for *i*th individual at time *j,* ${C}_{{ipred}_{ij}}$ is the individual predicted concentration for that observation, and ${\varepsilon }_{ij}$ is a random variable from a normal distribution with a mean of zero and a variance fixed to 1. SD assumes that the standard deviation of the residual error is the sum of the additive and proportional components. Separate ${SD}_{1}$ and ${SD}_{2}$ values were tested for each dataset, compound and for plasma and urine samples. If ${SD}_{2}$ was not significantly different than 0, then only the ${SD}_{1}$ term was kept, collapsing the error structure into a log-additive error model.

The selection of structural and stochastic models was based on objective function values (OFV) and goodness of fit plots. The difference in the OFV between 2 hierarchical models was assumed to follow a *χ*^2^ (chi-square) distribution and for 1 degree of freedom a decrease in OFV of 10.83, corresponding to a significance level (α) of 0.001, was taken to be statistically significant.

The following covariates were evaluated in our study: birthweight, current weight, gestational age (GA), PNA, postmenstrual age (PMA), sex, Z-score for birthweight [18], and being small for gestational age (SGA) [18]. As in dataset 1 current weight was not reported and all samples were collected within the first week of life, we assumed that current weight was equal to birthweight. No other covariate information was missing in dataset 1. In dataset 2, information on current weight was missing for some observations within a patient. In those cases, linear interpolation was used for current weight between two existing current weight measurements of the particular patient.

For implementation of continuous covariates (birthweight, current weight, GA, PNA, PMA, birthweight Z-score), linear, power, exponential, and sigmoidal functions were tested. For categorical covariates (gender, SGA), additive shift models were tested. Covariates were evaluated based on OFV by a stepwise forward inclusion (ΔOFV > 6.64, P < 0.01) and backward deletion (ΔOFV < 10.83, P > 0.001) process. Besides this, goodness of fit plots split for datasets and covariate quartiles, plots of individual eta-values *versus* covariate values (ETA plots), and reduction in inter-individual variability on the parameter of interest, were evaluated.

### Model Evaluation

A bootstrap analysis (n = 500) stratified on dataset, was performed to assess the robustness of the final model and parameter estimates. A normalized prediction distribution error (NPDEs) analysis was performed based on 1,000 simulations, to assess the predictive ability of the final model. Each observed concentration was compared to the range of simulated concentrations using the NPDE package in R, in output stratified for each compound and biological matrix of the measurement [19]. The NPDEs of urine samples were plotted together due to a limited number of samples per compound.

### Model Simulation

The final model was used to simulate PCM and metabolite concentrations under six-hourly intravenous PCM administration of 7.5 mg/kg for typical hypothetical preterm neonates with different birthweight and PNA. This was done to illustrate the concentration–time profiles of each compound under the current practice of linear bodyweight-based dosing. In addition, dosage adaptations were proposed aiming for average steady-state concentrations (Css_ave_) of PCM of around 9 mg/L [13, 20] across preterm neonates with different birthweight and PNA. In these simulations, the growth curves published by Anchieta *et al*. [21] were used to describe the postnatal changes in current weight for different birthweight neonates.

## Results

### Population Pharmacokinetics Model Development

In the analysis of PCM plasma samples without metabolites, a two-compartment model was identified with current weight as covariate for distribution volume of the central compartment, and birthweight and PNA as covariates for total PCM clearance. The combination of birthweight and PNA as covariates on CL was significantly better than current weight alone (ΔOFV = -93) and in case of the latter, a positive trend of ETA on CL with PNA and a negative trend with birthweight was observed. When current weight and PNA were combined instead of birthweight and PNA, OFV increased 33 points due to the over-prediction of PCM CL for neonates with lower birthweight. When PMA and current weight were used instead of birthweight and PNA, OFV increased by 79 points and there was still a positive trend in the ETA on CL *versus* PNA plots (figures not shown).

**Fig. 1:**
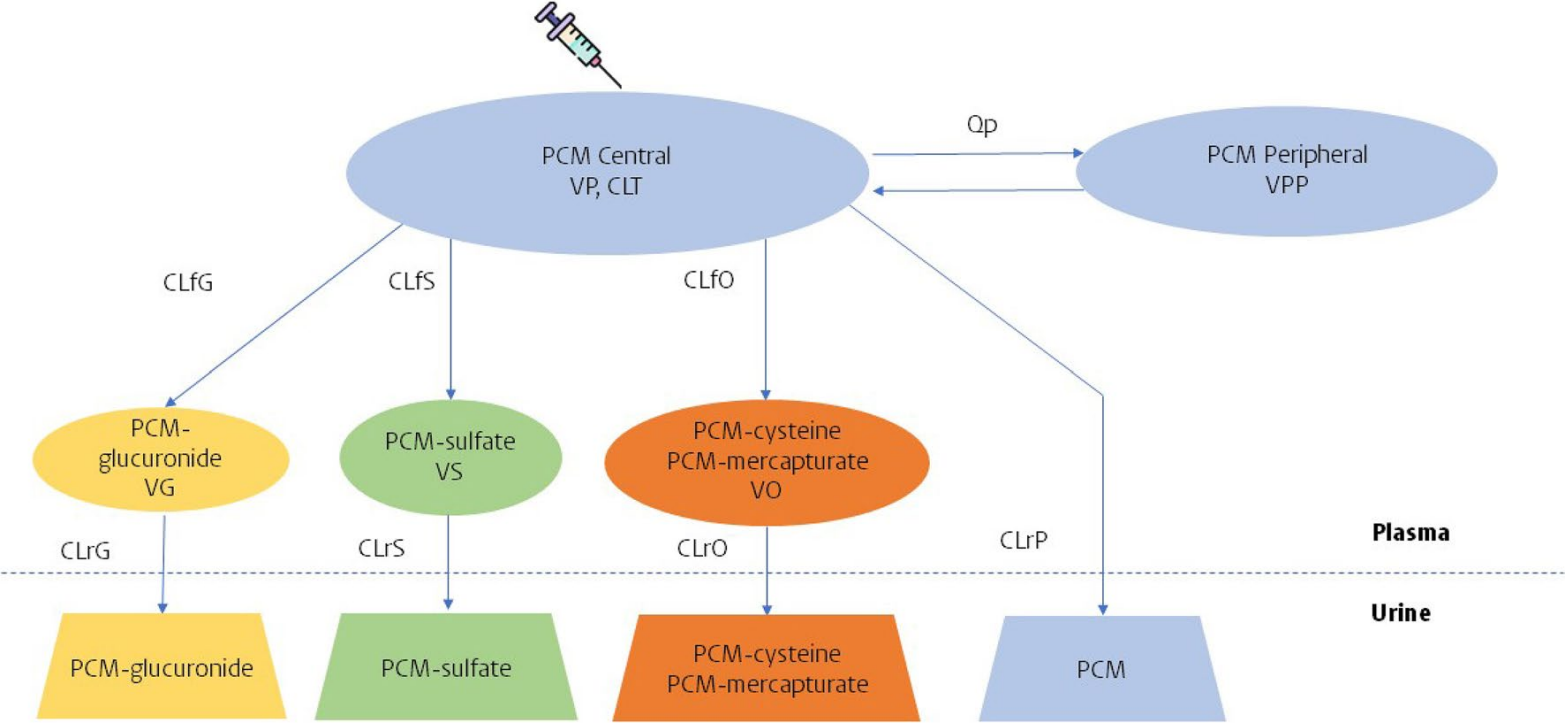
Schematic illustration of the final model of paracetamol (PCM) and its metabolites. CLfG, CLfS, CLfO are the formation clearance of PCM-glucuronide, PCM-sulfate, and the combined oxidative metabolites (PCM-cysteine and PCM-mercapturate), respectively; CLrP, CLrG, CLrS, CLrO are the renal clearance of PCM and its respective metabolites; CLT is the total PCM clearance and is the sum of CLfG, CLfS, CLfO and CLrP; Qp is the inter-compartmental clearance of PCM; VP, VG, VS, VO are the distribution volumes of the central compartment of PCM, PCM-glucuronide, PCM-sulfate and the combined oxidative metabolites, respectively; VPP is distribution volume of the peripheral compartment of PCM.

When including PCM and all metabolite concentrations in plasma and urine in the analysis, in addition to a two-compartment model for PCM, one-compartment models for each of the metabolites were found to describe the disposition of metabolites well. A schematic representation of the model structure of PCM and metabolites in plasma and urine is shown in Fig. 1.

It was observed that the sum of the predicted formation clearance of each elimination route of PCM, identified using the urine observations, was much smaller than the total PCM clearance identified using plasma samples, indicating a loss of urine volume in the collection process from the diaper. To address this, the obtained urine volume was multiplied by an estimated correction factor with inter-sample variability quantifying deviations from this correction factor among urine samples. The introduction of correction factor and its inter-sample variability reduced OFV of 388 points (*p* < 0.001) and the correction factor was estimated to be 3.6 with inter-sample variability of 138.4%.

In the covariate analysis, the ETA plots showed a negative relationship for sulfation (*R*_*FS*_) with PNA and a positive trend for glucuronidation (*R*_*FG*_) and oxidation (*R*_*FO*_) with PNA (Eq. 1). PNA was included in the model on *R*_*FS*_ using a sigmoidal function (ΔOFV = -55, *P* < 0.001):

8 $$R_{FS}=R_{{FS}_0}\times\left(1-\frac{PNA}{{PNA}_{50}+PNA}\right)$$

where ${R}_{FS}$ is the fold difference of sulfation CL relative to the unchanged renal clearance of PCM (see methods), ${R}_{{FS}_{0}}$ is the fold difference at birth, and ${PNA}_{50}$ is the PNA at which ${R}_{FS}$ reaches half of ${R}_{{FS}_{0}}$. A result of this implementation of a negative influence of PNA on sulfation through ${R}_{FS}$, was a subsequent increase with PNA in the fraction of glucuronidation (*FG*)$,$ and oxidation (*FOX*), as a consequence of the parametrization as expressed by Eq. 1 (see methods). Additionally, a slight trend of increasing ${R}_{FG}$ with increasing birthweight was observed, for which a power function was found to best describe this relationship (ΔOFV = 4.16, *p* < 0.05).

During the covariate analysis on the renal elimination clearance of metabolites, birthweight in a power function in combination with PNA in a linear relationship was found to best describe their renal clearance. Therefore, the same covariate relationship with birthweight and PNA and the same random effect on renal elimination clearance, across all compounds, were used, while keeping the typical CL values estimated for each pathway to account for the different fractions of plasma binding and/or reabsorption of the different compounds. This implementation is based on the idea that these compounds are all renally excreted and that therefore the maturation pattern and thereby their covariate relationship will most likely be similar. Compared to separate estimation of covariate relationship for each compound, this model simplification only increased OFV by 26 points while reducing the number of parameters by 4.

For the residual variability, separate SD terms were used for each dataset, compound in the plasma samples. A shared SD term was used for all metabolites in urine due to the limited number of urine samples. The parameters of the final model are presented in Table II. NONMEM code for the final model is provided in the Supplementary Materials.

**Table II:** Parameter Estimates of the Final Model

Parameter			Parameter estimate (RSE %)	IIV as CV%* (RSE%) [shrinkage %]	Bootstrap	
					Parameter estimate (95% CI)	IIV as CV%* (95% CI)
PCM						
$$\mathrm{CLT }(\mathrm{L}/\mathrm{h}) =\mathrm{TVCLT}\times {(BWb/985)}^{{\theta }_{BWb}}\times (1+(PNA-5)\times {\theta }_{PNA})$$						
TVCLT			0.137 (3)	30.9 (7) [11]	0.137 (0.129–0.146)	30.1 (24.2–36.7)
θ_BWb_			0.902 (11)		0.903 (0.686–1.10)	
θ_PNA_			0.0469 (12)		0.0464 (0.0310–0.0610)	
VP (L) = $\mathrm{TVVP}\times {(BWc/1012)}^{{\theta }_{BWc}}$						
TVVP			0.955 (3)	28.3 (8) [14]	0.955 (0.775–1.030)	28.1 (20–39.9)
θ_BWc_			0.836 (11)		0.844 (0.659–1.05)	
Qp			0.0494 (19)		0.0464 (0.0249–0.508)	
VPP			0.244 (12)		0.247 (0.167–0.348)	
Volume of metabolites						
$VG\left(L\right)=fVG\times VP$ $VS\left(L\right)=fVS\times VP$ $VO\left(L\right)=fVO\times VP$						
fVG			0.6 (9)		0.6 (0.5–0.8)	
fVS			0.326 (4)		0.330 (0.300–0.417)	
fVO			0.807 (6)		0.802 (0.699–1.01)	
Renal clearance of metabolites						
$\mathrm{CLrG }(\mathrm{L}/\mathrm{h})=TVCLrG\times {(BWb/985)}^{{\theta }_{BWb}}\times (1+(PNA-5)\times {\theta }_{PNA})$ $\mathrm{CLrS }(\mathrm{L}/\mathrm{h})=TVCLrS\times {\left(BWb/985\right)}^{{\theta }_{BWb}}\times \left(1+\left(PNA-5\right)\times {\theta }_{PNA}\right)$ $\mathrm{CLrO }(\mathrm{L}/\mathrm{h})=TVCLrO\times {(BWb/985)}^{{\theta }_{BWb}}\times (1+(PNA-5)\times {\theta }_{PNA})$						
TVCLrG			0.0425 (5)	37.6 (9) [16]	0.0428 (0.0383–0.0482)	36.8 (28.8–49.3)
TVCLrS			0.0256 (4)		0.0258 (0.0233–0.0286)	
TVCLrO			0.0498 (5)		0.0499 (0.0446–0.0561)	
θ_BWb_			1.62 (9)		1.61 (1.30–1.95)	
θ_PNA_			0.0854 (12)		0.0830 (0.0578–0.120)	
Formation clearance of metabolites						
$CLfG (L/h)={R}_{FG}/({R}_{FS}+{R}_{FG}+{R}_{FO}+1)\times CLT$ $CLfS (L/h)={R}_{FS}/({R}_{FS}+{R}_{FG}+{R}_{FO}+1)\times CLT$ $CLfO (L/h)={R}_{FO}/({R}_{FS}+{R}_{FG}+{R}_{FO}+1)\times CLT$ ${\mathrm{R}}_{\mathrm{FG}}={\mathrm{TVR}}_{\mathrm{FG}}\times {\left(BWb/985\right)}^{{\theta }_{BWb}}$ ${\mathrm{R}}_{\mathrm{FS}}={\mathrm{TVR}}_{\mathrm{FS}}\times (1-\mathrm{PNA}/({\theta }_{\mathrm{PNA}50}+\mathrm{PNA})$ )						
${\mathrm{TVR}}_{\mathrm{FG}}$			1.16 (9)	58.1 (9) [23]	1.15 (0.899–1.52)	56.5 (43.3–71.5)
${\theta }_{BWb}$			0.479 (45)		0.487 (0.137–0.917)	
${\mathrm{TVR}}_{\mathrm{FS}}$			24.5 (11)	44.5 (10) [26]	25.4 (18.4–35.4)	42.7 (31–56.8)
${\theta }_{\mathrm{PNA}50}$			9.18 (23)		8.71 (4.55–15.6)	
${R}_{FO}$			3.51 (8)	62.7 (8) [15]	3.48 (2.65–4.54)	60.8 (46.6–78.8)
Urine volume correction factor			3.62 (16)	138.4 (11) [44]	3.62 (2.69–5.30)	132.2 (82.9–206.8)
Parameters			Parameter estimate (RSE %)	Combined shrinkage %	Bootstrap	
Plasma	Dataset 1 SD1	PCM	0.166 (6)	14	0.164 (0.132–0.202)	
		PCM-glucuronide	0.325 (6)		0.320 (0.256–0.382)	
		PCM-sulfate	0.168 (10)		0.159 (0.098–0.212)	
		PCM-oxidative pathway metabolites	0.208 (11)		0.201 (0.142–0.277)	
	Dataset 2 SD1	PCM	0.345 (5)		0.342 (0.280–0.399)	
		PCM-glucuronide	0.858 (5)		0.842 (0.653–1.05)	
		PCM-sulfate	0.325 (6)		0.322 (0.266–0.371)	
		PCM-oxidative metabolites	0.369 (5)		0.359 (0.285–0.443)	
	Dataset 1 SD2	PCM-sulfate	0.464 (16)		0.457 (0.268–0.841)	
		PCM-oxidative metabolites	0.0489 (22)		0.0507 (0.0212–0.0850)	
Urine	Dataset 1 SD1	Combined error	0.115 (26)		0.106 (0.0436–0.291)	

^*^CV% is calculated by sqrt(exp(estimate)-1)*100%

BWb, birthweight (g); BWc, current weight (g); CI, confidence interval; CLfG, CLfS, CLfO, the formation clearance of PCM-glucuronide, PCM-sulfate, and the combined oxidative metabolites (PCM-cysteine and PCM-mercapturate), respectively; CV, coefficient of variation; RSE, relative stand error; IIV, inter-individual variability; PCM, paracetamol; PNA, postnatal age (days); R_FG_, R_FS_, R_FO_, the fold difference of the formation clearance of PCM-glucuronide, PCM-sulfate, and the combined oxidative metabolites (PCM-cysteine and PCM-mercapturate) relative to the renal clearance of unchanged PCM; CLT, total PCM clearance; CLrG, CLrS, CLrO, the renal clearance of PCM-glucuronide, PCM-sulfate, and the combined oxidative metabolites (PCM-cysteine and PCM-mercapturate); Qp is the inter-compartmental clearance of PCM; SD, standard deviation; VP, VG, VS, VO are the distribution volumes of the central compartment of PCM, PCM-glucuronide, PCM-sulfate and the combined oxidative metabolites, respectively; VPP, is distribution volume of the peripheral compartment of PCM

**Fig. 2:**
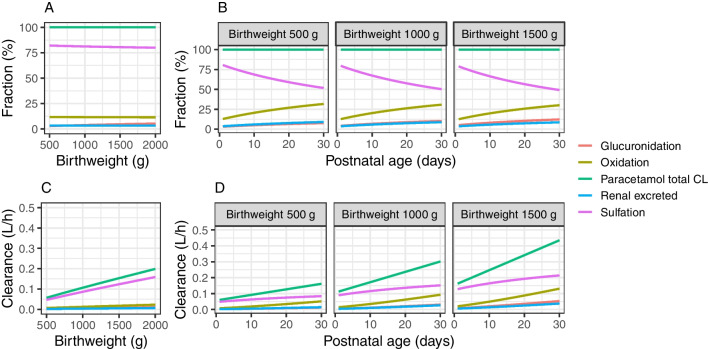
Fraction of total paracetamol clearance for the different elimination pathways (upper panels **A** and **B**) and absolute clearance values of the different elimination pathways of paracetamol (lower panels **C** and **D**) *versus* birthweight (left panels **A** and **C**) and *versus* postnatal age for different birthweights (right panels **B** and **D**) according to the final model.

Figure 2 illustrates how the fractions of total PCM clearance (A, B) and absolute clearance values through the different elimination pathways (C, D) vary with birthweight and PNA. This illustrates, amongst others that total PCM CL at birth in neonates with a birthweight of 1,500 g is 169% higher compared to neonates of 500 g. For glucuronidation, oxidation and sulfation, these percentages are 347%, 164% and 164%, respectively. When PNA increases from 1 to 30 days, the total PCM CL is predicted to increase 167%, with around 550% increase in formation CL for PCM-glucuronide and the oxidative metabolites, and 70% increase in formation of PCM-sulfate; *e.g.,* for a neonate with a birthweight of 500 g, as PNA increases from day 1 to day 30, total PCM CL increases from 0.06 to 0.16 L/h. This increase is attributed to increase in glucuronidation (0.0018 to 0.012 L/h), sulfation (0.049 to 0.084 L/h), and oxidation (0.0077 to 0.051 L/h). The corresponding increase for a 1,500 g neonate is 0.16 to 0.43 for total PCM CL, attributed to increase in glucuronidation (0.0082 to 0.053 L/h), sulfation (0.13 to 0.21 L/h), and oxidation (0.02 to 0.13 L/h). The relatively small increase in the absolute formation CL of PCM-sulfate compared to other pathways leads to a decrease in its contribution to the total PCM clearance as PNA increases.

### Model Evaluation

The goodness-of-fit plots of plasma and urine samples (Supplementary Fig. S2, S3 and S4) indicate that the final model describes the plasma concentrations and excreted amounts of PCM and its metabolites well. The bootstrap results (Table II, convergence rate 95.8%) showed that the bootstrap estimates were all within ± 10% of the parameter estimates of the final model, underlining the robustness of the final model. No structural trends were observed in NPDEs when plotted against TAD, birthweight, GA, current weight, and model predicted concentrations, even though a slightly over prediction of the variability was seen (Supplementary Fig. S5).

### Model Simulations

**Fig. 3:**
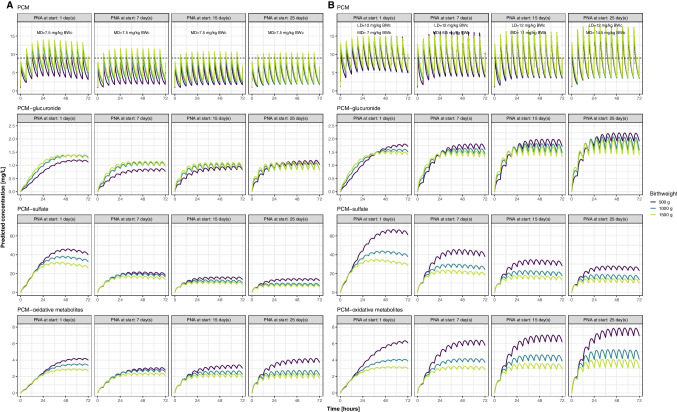
Plasma concentrations of paracetamol (PCM), PCM-glucuronide, PCM-sulfate, and the combined PCM oxidative metabolites (PCM-cysteine and PCM-mercapturate) after intravenous PCM of 7.5 mg/kg per 6 h (**A**, left panels) or birthweight and PNA based dosing according to Table III (**B**, right panels) for three days for typical neonates with different birthweight and PNA at start of dosing according to the final model. Note the concentrations of metabolites are PCM equivalent concentrations. BWb, birthweight; BWc, current weight; LD, loading dose; MD, maintenance dose.

Based on the final model, the population predicted plasma concentration of PCM and its metabolites for typical neonates under six-hourly intravenous PCM of 7.5 mg/kg, are plotted in Fig. 3A. Using this dosage, PCM concentrations decrease with increasing PNA as a result of CL values that increase more rapidly with PNA than the linear bodyweight dosing accounts for.

In order to match PCM exposure across the population, we propose a new birthweight and PNA adjusted dosing regimen with a loading dose of 12 mg/kg in Table III. Concentrations obtained with this regimen are plotted in Fig. 3B. The figure shows that this model-derived regimen results in more consistent PCM exposure across the heterogeneous population of preterm neonates represented in our dataset (upper panels of Fig. 3B *versus* Fig. 3A). For the metabolites, concentrations among the representative typical individuals vary according to the combined impact of formation clearance and renal elimination clearance of each metabolite. PCM-glucuronide concentrations (Fig. 3) show modest changes with birthweight and PNA in both regimens, indicating the rough offset in the changes from formation rate and from elimination rate. PCM-sulfate concentration is the highest for lower BW and lower PNA and decreases considerably with PNA as a result of the increasing renal clearance overtaking the slow increase of formation clearance. PCM-oxidative metabolites concentrations increase with PNA and neonates with lower birthweight have the highest concentrations, due to lower renal elimination CL in this population (Fig. 3B). As current weight is more often used in the clinics, we also simulated a regimen where the dose according to Table III is given, but the maintenance dose was based on current weight and PNA instead of birthweight and PNA (supplementary Fig. S6(A)). The plot shows that the Css_ave_ for the first 15 days will be lower than the birthweight based dosing. Also, there is larger difference in concentrations between neonates with different birthweight.

**Table III:** Model-Derived Paracetamol Dosing Regimen for Preterm Newborns < 32 Weeks of Gestation for the First Month of Life

Postnatal age (days)	Loading dose (mg/kg current weight)	Maintenance dose Dose per 6 h (mg/kg birthweight)
0–3	12	7
4–9		8.5
10–19		11
20–30		14.5

## Disscussion

Our study characterizes the concentration–time profile of PCM and its metabolites in preterm neonates below 32 weeks of gestation, with specific emphasis on the quantification of the maturation patterns of the different pathways. The combined two datasets provided plasma and urine samples after intravenous PCM dosing with rich sampling upon single dose and sparse sampling upon multiple dosing, ultimately enabling the characterization of the bi-exponential elimination of PCM and the identifiability of the metabolite model. Additionally, the second dataset provided more information on the maturation trajectory as it included neonates with higher PNA. Based on all data, birthweight and PNA, as indicators for prenatal and postnatal maturation respectively, were identified as significant determinants of maturational change in CL of PCM and its elimination pathways. As such, this analysis provides insight in the prenatal and postnatal maturation of PCM CL and its metabolites in this population, which may ultimately also facilitate the description of PK of other drugs that share the same pathway.

Glucuronidation of PCM is mainly catalyzed by UDP glucuronosyltransferase (UGT) isoenzymes 1A1, 1A6, and 1A9 [22]. Although glucuronidation is the main elimination pathway in adults (55%), our study showed that its contribution in neonates is relatively small (5–15%), in line with previous PK studies [9, 12, 23, 24]. The low glucuronidation activity after birth, specifically for UGT1A1, is the well-known cause of unconjugated hyperbilirubinemia in newborns after preterm birth [25]. Glucuronidation of PCM increases with increasing birthweight, leading to an increased glucuronide–to–sulfate (G:S) metabolite ratio (the ratio ranges from 0.03 to 0.06 for birthweights between 500 and 1,500 g), similar to previous reports [12, 24]. In addition to that, glucuronidation of PCM increases considerably with PNA, with a predicted sixfold difference between day 1 and day 30. The maturation of glucuronidation is expected to be completed later in childhood, as its contribution is still small in our research population. Bhatt *et al*. [26] showed that the abundances of UGT1A1, 1A6 and 1A9 per mg microsomal protein increase 8, 55 and 35-fold respectively, from neonates to adults, and although this may not directly translate into the same fold-change in CL, it does show a considerable increase in its metabolic capacity.

Sulfation is the dominant elimination pathway of PCM in neonates and is mainly carried out by sulfotransferase (SULT) SULT1A1, 1A3/4, 2A1, and 1E1 [27, 28]. Our study showed that sulfation slowly matures antenatally in our population, *i.e.,* between 24 and 32 weeks of gestation (Fig. 2). This slow increase in activity follows a dramatic activity reduction around 20–22 weeks of gestation, according to reported *in vitro* activity studies. This decrease in the early gestation is probably caused by decreased SULT1A3/4 and SULT1E1 activities whereas the increase in later gestation and postnatal life represents increases in SULT2A1 [28, 29]. After birth, sulfation CL shows slow maturation. This is confirmed by the study of Cook *et al*. [9], in which current weight alone was used to describe the increases in formation CL of PCM-sulfate.

Adult studies [4, 30] have reported an increase of glucuronide–to–sulfate (G:S) metabolite ratio after repeated dosing. Provided explanations for this observation included upregulated UGT activity or saturated sulfation [4, 31–33]. In neonates, only one study [23] has reported an increased urine PCM-glucuronide to total PCM excretion ratio after multiple dosing. However, this was later suggested to be the result of increasing urine excretion rate of PCM-glucuronide as its concentration increases over time before steady-state is reached without changes in CL [7]. Studying the impact of repeated dosing on metabolic pathways in neonates is difficult with maturation as a potential confounder and the high between-subject variability. Our study also could not identify a significant influence of multiple dosing in preterm neonates on either formation CL of glucuronidation or of sulfation.

CYP2E1 is the main isoenzyme responsible for the conversion of PCM to N-acetyl-p-benzoquinone imine (NAPQI) [34]. Hepatic expression of CYP2E1 is reported to increase slowly during the prenatal period and then increases rapidly after birth [35], which is in accordance with our findings (Fig. 2). The oxidation pathway takes up around 10–25% of total PCM CL in neonates and the percentage is larger compared to adults (5–10%). Johnsrud *et al*. [35] reported that the expression of CYP2E1 per mg microsomal protein approaches adult values by approximately 90 days of PNA, suggesting the maturation of its activity completes early in childhood.

Hepatoxicity occurs when the toxic intermediate metabolite NAPQI fails to bind to the cysteine residue of glutathione, after the former’s production rate exceeds the regeneration rate of glutathione. As a result, excess NAPQI conjugates hepatic macromolecules, initiating hepatotoxicity [36]. In such cases, N-Acetylcysteine can be used for detoxification [37]. Compared to adults, the absolute formation CL of the oxidation pathway in neonates is lower. Furthermore, it is generally assumed that neonates exhibit faster glutathione synthesis which leads to higher glutathione conjugation capacity. As a result, serious hepatotoxicity or death following acute PCM overdose is rarely reported in neonates [1, 37, 38]. Roofthooft *et al*. [39] also showed that for dosages of 15 mg/kg/6 h intravenously for 3–7 days in extremely preterm neonates for closure of patent ductus arteriosus, no abnormal liver function is observed. In theory, metabolites of the CYP2E1 pathway (PCM-cysteine and PCM-mercapturate) would show a non-linear formation if there is excess NAPQI that cannot be conjugated, but our study could not identify this non-linearity among the dosages given in this study.

In our study, changes in total PCM CL are described using birthweight and PNA. This is different from most of the previous studies in preterm and term neonates [9–11], in which current weight is mostly identified as descriptor of maturation. One possible reason is that our study focuses on extremely to very preterm neonates, and, with larger amounts of data, can detect the details in maturation that cannot simply be described by current weight alone. As a result, linear bodyweight-based dosing is not ideal for this population and can lead to PCM concentration difference across individuals with different birthweight and PNA (Fig. 3A). Therefore, in order to achieve the same PCM exposure in this very premature population, it should be considered to adjust the dose based on both birthweight and PNA. This study provides dosage adjustment for PNA below 30 days (Table III). Although the median PNA of our population is 5 days, there are sufficient PCM observations to support the modeling and therefore dosing regimen until 30 days of PNA (71 PCM observations for PNA between 10–20 days and 52 observations for PNA between 20–30 days). It is important to note that the target concentration used in this study is based on limited efficacy and short-term safety studies in neonates [20, 40], and there are still concerns regarding long term-safety of PCM for prenatal and neonatal use [41]. In addition, the high concentration of PCM-sulfate in the most immature neonates may be of concern, as studies have reported the depletion of sulfated sex hormones after using paracetamol [41, 42].

Our study used a correction factor to account for the possible urine volume loss during collection. The estimate of 3.6, along with its large variability, indicates the challenges and inaccuracy associated with urine collection in preterm neonates, particularly from diapers (issues like stool contamination, and remaining volume in the diaper, etc*.*). Still, thanks to the correction factor, we were able to quantify the individual loss and use information in the urine samples, such as the difference in the different metabolite amounts within a urine sample, and estimate the distribution volume of metabolites, achieving values similar to those reported by Cook *et al*. [9]. Nevertheless, the urine samples still showed higher amount variability that covered the possible covariate information. As a result, we made the assumption that the distribution volume of metabolites shared the same covariate relationship as PCM. Given that the bodyweight range in this study is not large, the impact of this assumption should be limited. However, no extrapolation of these results beyond the studied range in age and weight of these preterm neonates should be performed. This is also related to one limitation of this study, which only described the PCM PK until first 30 days of life. In order to be able to provide dosing guideline after first month for this population, population PK studies with more data beyond this age and physiologically-based PK analysis can be implemented. In addition, large interindividual variability in the formation CL of glucuronidation and oxidation still exists that cannot solely be explained by maturational factors. Possible explanations like genetic polymorphisms, comorbidity and environmental factors [6, 43] are beyond the scope of this study.

## Conclusions

In neonates born before 32 weeks of gestation, birthweight and PNA are important predictors for total PCM clearance and its glucuronidation, sulfation and oxidation. Total PCM CL increases with birthweight, with differences in fractions between the different pathways. After birth, there are considerable PNA related changes in the different elimination pathways, with significant increases in the absolute glucuronidation, oxidation and unchanged renal excretion CL of PCM, and modest increase in sulfation CL, resulting an increasing fraction of glucuronidation and oxidation, and a decreasing fraction of sulfation with PNA. Linear bodyweight-based dosing is not suitable to compensate for the large PNA-related maturation in these very immature preterm neonates when aiming to achieve similar PCM concentrations. Therefore, this study proposes a birthweight and PNA-based dosing regimen. Still, additional studies are needed to support the efficacy and long-term safety of this dosage.

### Supplementary Information

Below is the link to the electronic supplementary material.

Supplementary file1 (DOCX 371 KB)
