# Supplementary material for: Maturation of Paracetamol Elimination Routes in Preterm Neonates Born Below 32 Weeks of Gestation
Source: Pharm Res. 2023 Aug 21;40(9):2155–66. doi: 10.1007/s11095-023-03580-3 (PMC10547636; doi:10.1007/s11095-023-03580-3)
Supplement: Supplementary file 1 — Supplementary file1 (DOCX 371 KB) [file 11095_2023_3580_MOESM1_ESM.docx]

**Maturation of paracetamol elimination routes in preterm neonates born** **below 32 weeks of gestation**

**Supplementary materials**

Yunjiao Wu (1), Swantje Völler (1), Elke H.J. Krekels (1), Daniëlla W.E. Roofthooft (2), Sinno H.P. Simons (2), Dick Tibboel (3), Robert B. Flint (2,4), Catherijne A.J. Knibbe (1,2,5)*

(1) *Division of* *Systems Pharmacology and Pharmacy, Leiden Academic Centre for Drug Research, Leiden University, Leiden, The Netherlands.*

*(2). Department of Pediatrics, Division of Neonatology, Erasmus MC Sophia Children's Hospital, Rotterdam, The Netherlands*

*(3) Department of Pediatric Surgery, Erasmus University MC–Sophia Children’s Hospital, Rotterdam, The Netherlands*

*(4) Department of Hospital Pharmacy, Erasmus University Medical Center, Rotterdam, The Netherlands*

*(5) Department of Clinical Pharmacy, St Antonius Hospital, Nieuwegein, The Netherlands.*

***Corresponding author**

Prof. Catherijne A.J. Knibbe, PharmD, PhD, FCP

St. Antonius Hospital, Dept of Clinical Pharmacy

PO Box 2500

3430 EM Nieuwegein

The Netherlands

T 31 88 - 320 7252 or 72 25

F 31 88 - 320 72 49

E c.knibbe@antoniusziekenhuis.nl

**
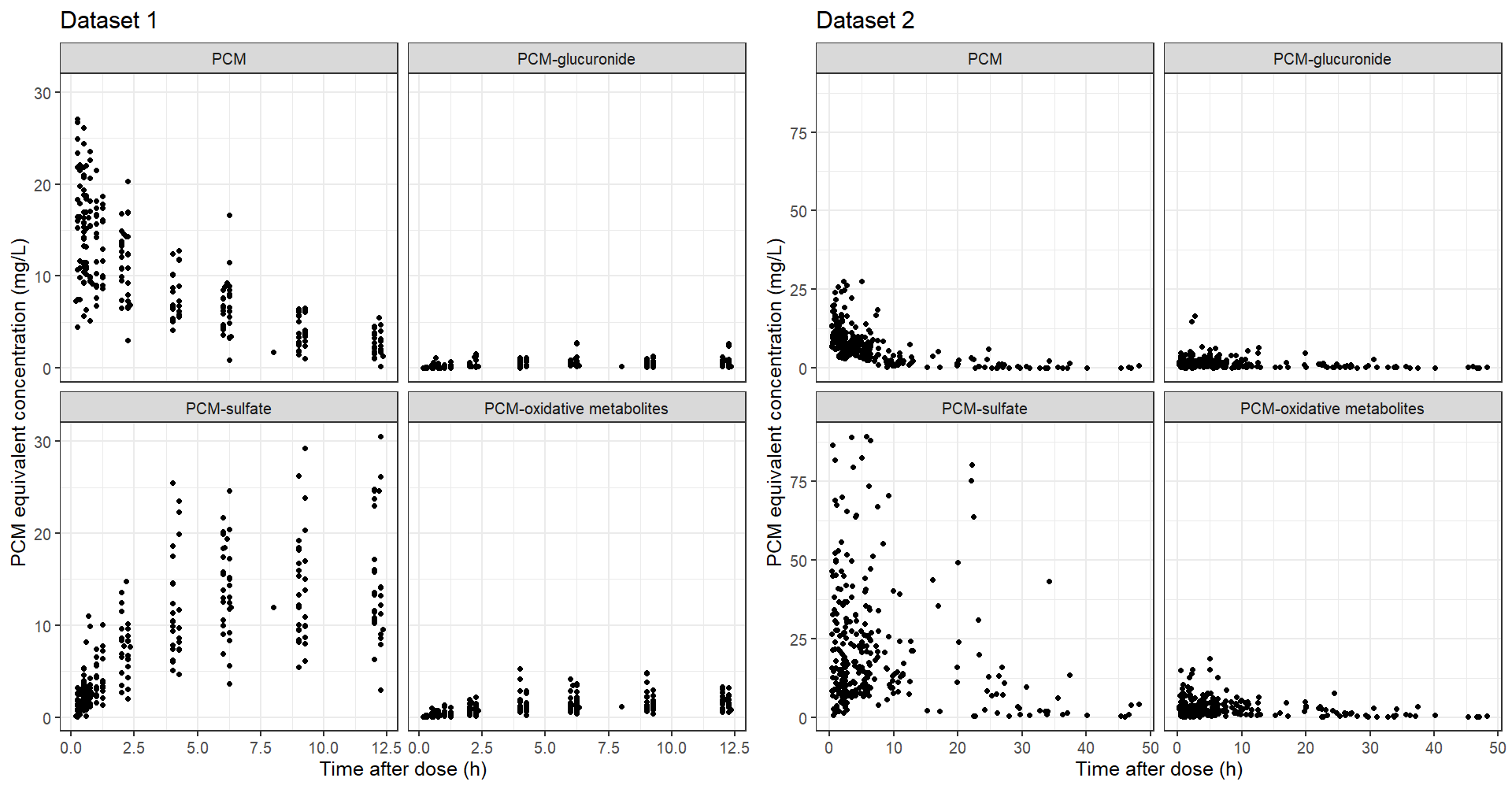
**

**Fig S1** Observed plasma concentration versus time after last dose (TAD) plot for paracetamol (PCM) and its metabolites split for dataset.


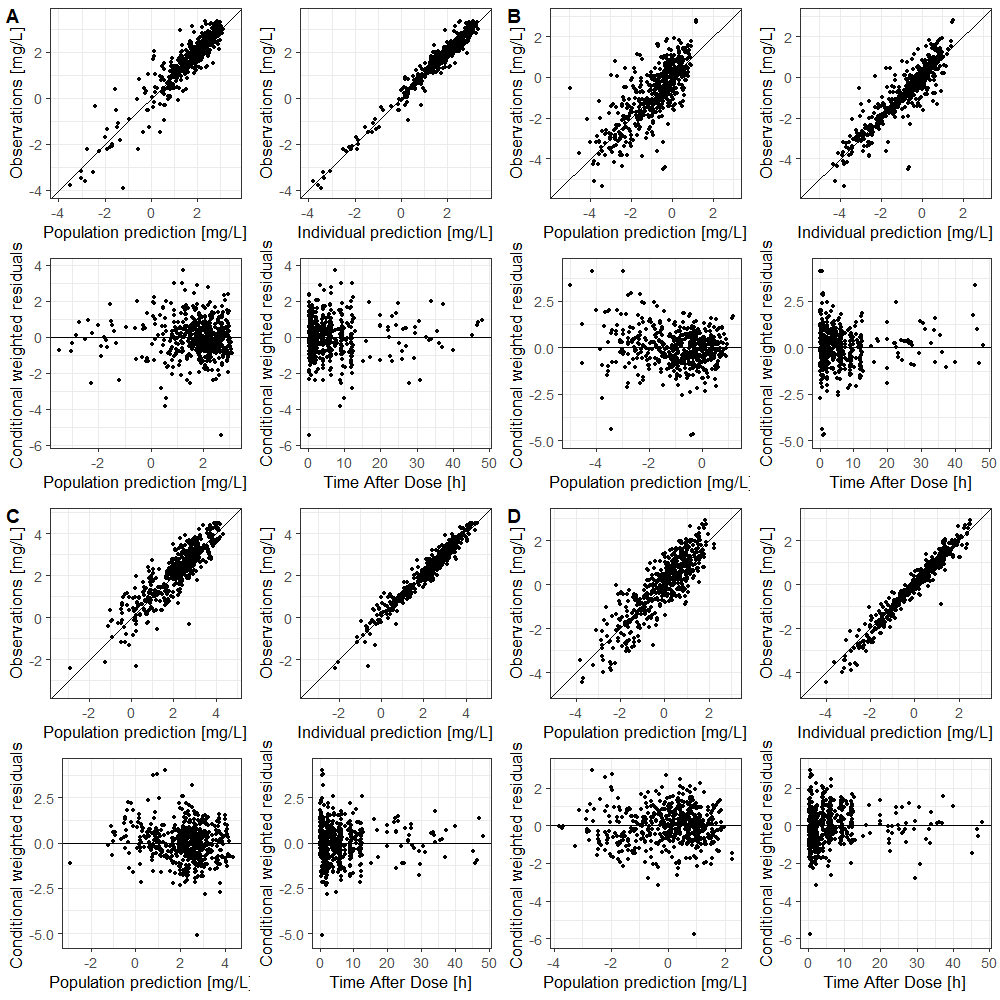


**Fig S2** Goodness-of-fit plots for log-transformed plasma concentrations of paracetamol (A), paracetamol-glucuronide (B), paracetamol-sulfate (C) and paracetamol oxidative metabolites (D)


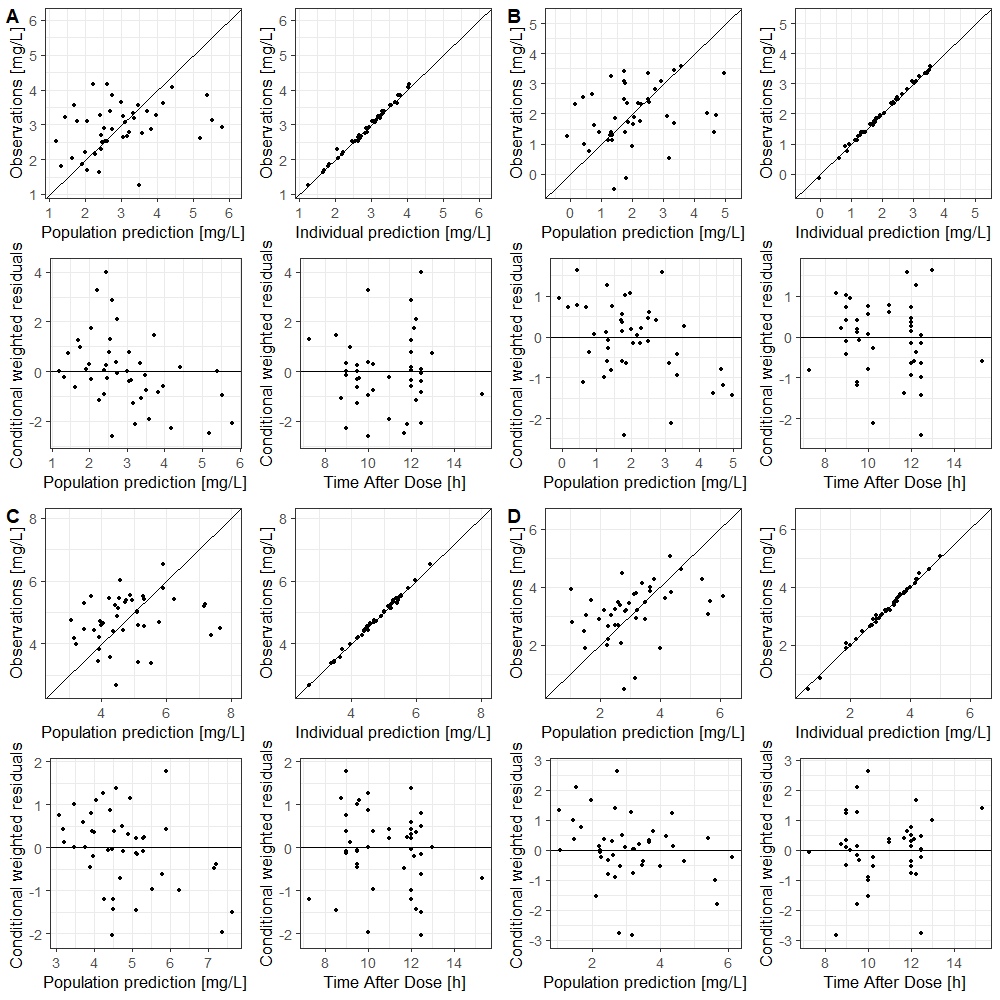


**Fig S3** Goodness-of-fit plots for log-transformed urine concentrations of paracetamol (A), paracetamol-glucuronide (B), paracetamol-sulfate (C) and paracetamol oxidative metabolites (D)


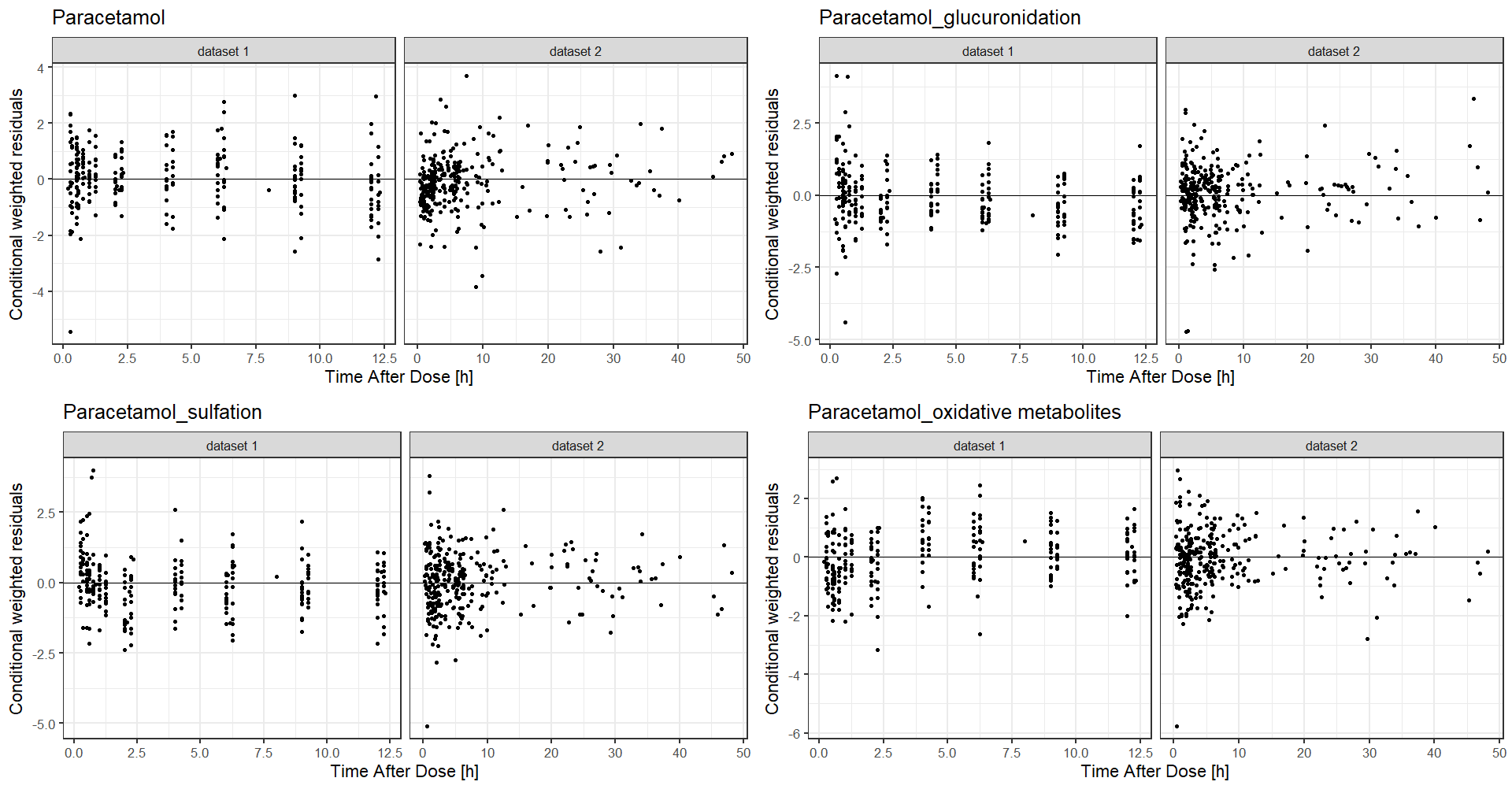


**Fig S4** Conditional weighted residuals versus time after dose plots for plasma concentrations of paracetamol, paracetamol-glucuronide , paracetamol-sulfate and paracetamol oxidative metabolites, split by dataset.


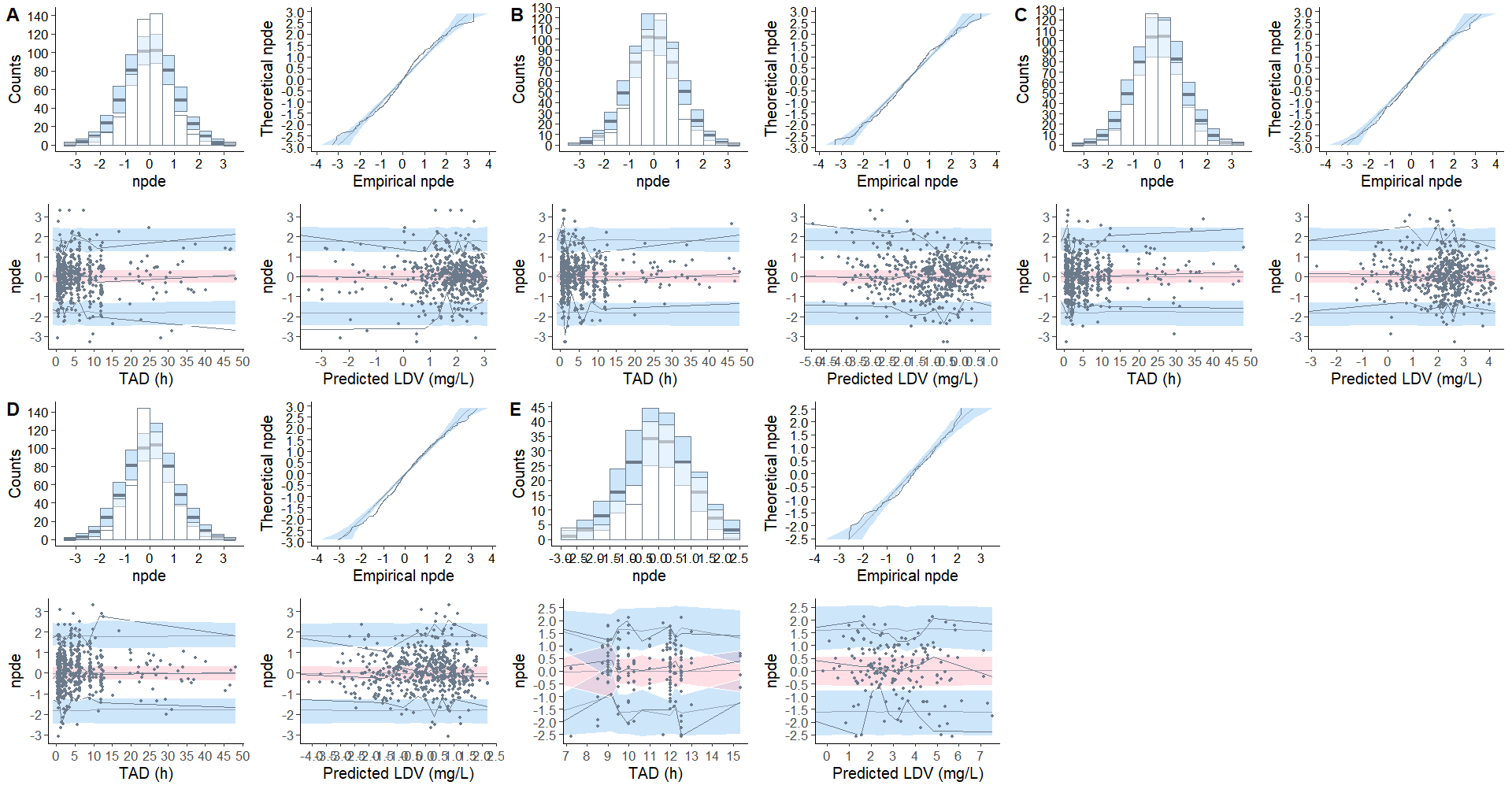


**Fig S5** Normalized Prediction Distribution Errors (NPDE) results for plasma concentrations of paracetamol (A), paracetamol-glucuronide (B), paracetamol-sulfate (C) and paracetamol oxidative metabolites (D) and the urine concentrations of all compounds (E). For each compound, upper two plot are the distribution of NPDE shown as a histogram (left) and a QQ-plot (right) with blue area representing the 90% prediction interval under the theoretical N(0,1) distribution. Bottom two plots are the scatterplots of NPDE versus time after last dose (TAD) (left) and NPDE versus model predictions (right). Dots represents the NPDE computed for the dataset. The lines show the evolution of three empirical percentiles (2.5, 50 and 97.5) for the observed data (dark grey) compared to the model predictions (light grey). The pink band corresponds to the prediction interval for the median of the NPDE (50th percentile) and the blue bands the prediction intervals for the 2.5 and 97.5th percentiles.


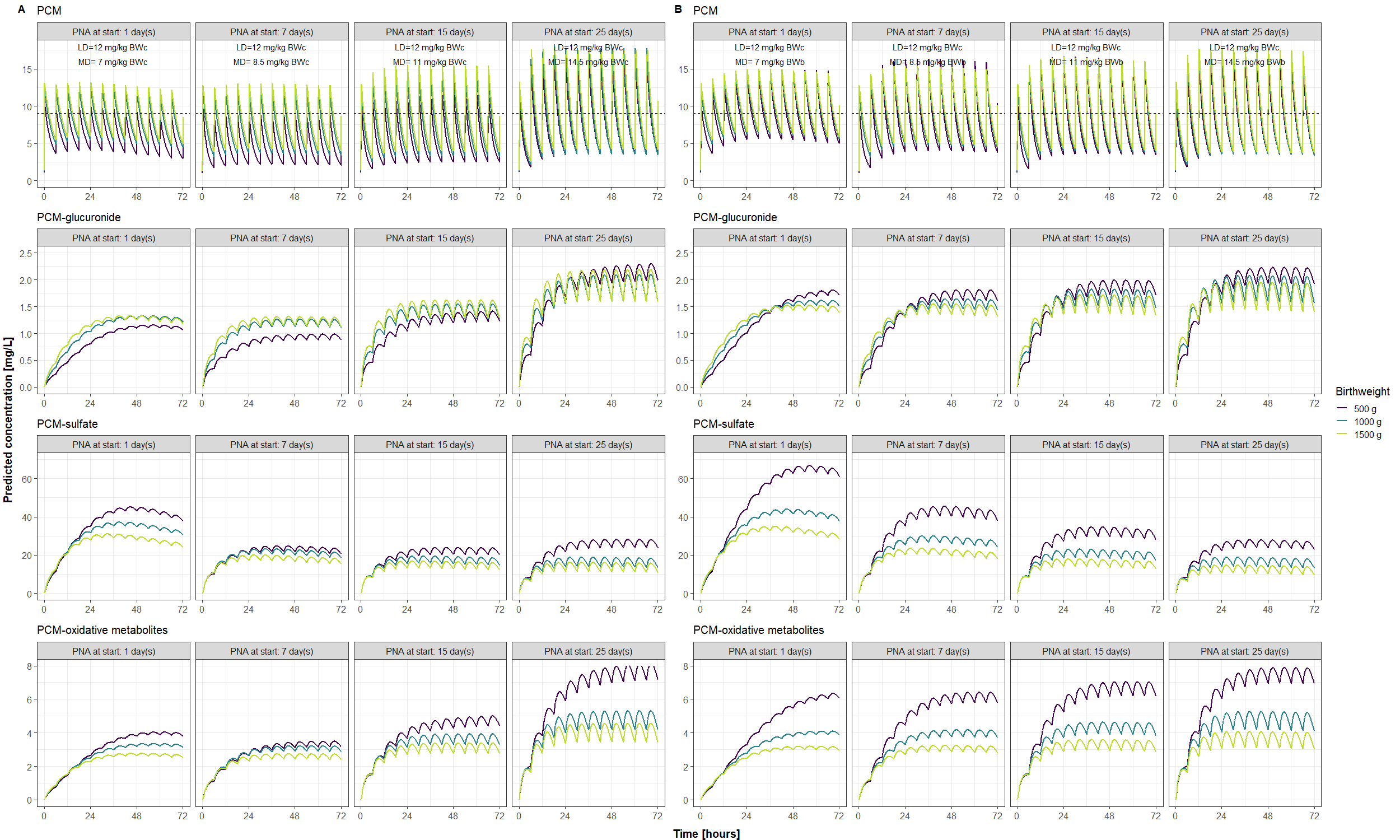


**Fig S6** Plasma concentrations of paracetamol (PCM), PCM-glucuronide, PCM-sulfate, and the combined PCM oxidative metabolites (PCM-cysteine and PCM-mercapturate) after dosing according to Table 3 when current bodyweight is used (A, left panel) compared to when birthweight is used (B, right panel), for three days for typical neonates with different birthweight and PNA at start of dosing according to the final model. Note the concentrations of metabolites are PCM equivalent concentrations. BWb, birthweight; BWc, current bodyweight; LD, loading dose; MD, maintenance dose.

**NONMEM® Control Stream for the Final Population Pharmacokinetic Model**

$PROBLEM PK

$INPUT O ID TIME AMT RATE INF EVID MDV ODV WT PNA TAD BLOQ BW GA SEX PMA MDIFF SGA CMT ULOQ ASY DV ZSCORE NDOSE OCC UVOL

$DATA 20220621CombineUrine_final.csv IGNORE=@ IGNORE(PNA.GT.30) IGNORE(BLOQ.EQ.2);;

$SUBROUTINES ADVAN6 TOL=6

$MODEL

NCOMPARTMENTS= 9

COMP= (CENTPCM) ; Central compartment for paracetamol in plasma

COMP= (CENTGLUC) ; Central compartment for paracetamol-glucuronide in plasma

COMP=(CENTSULF) ; Central compartment for paracetamol-sulfate in plasma

COMP=(CENTOXI) ; Central compartment for paracetamol-oxidative metabolites in plasma

COMP= (URINEPCM INITIALOFF) ; Central compartment for paracetamol in urine

COMP= (URINEGLUC INITIALOFF) ; Central compartment for paracetamol-glucuronide in urine

COMP=(URINESULF INITIALOFF) ; Central compartment for paracetamol-sulfate in urine

COMP=(URINEOXI INITIALOFF) ; Central compartment for paracetamol-oxidative metabolites in urine

COMP = (PERIPH) ; Peripheral compartment for paracetamol in plasma

$PK

;Paracetamol parameters

TVCL1 = THETA(1) *(BW/985)**THETA(5) *(1+(PNA-5)*THETA(6))

TVV1 = THETA(2)* (WT/1012)**THETA(7)

TVV9 = THETA(3)

TVQ = THETA(4)

CL1 = TVCL1* EXP(ETA(1)) ;Total CL of paracetamol

V1 = TVV1 * EXP(ETA(2)) ;Volume of distribution of central compartment of paracetamol

S1 = V1

V9 = TVV9 ;Volume of distribution of peripheral compartment of paracetamol

S9 = V9

Q = TVQ ;Intercompartment CL of paracetamol

;Paracetamol-glucuronide parameters

TVCL2= THETA(8)*(BW/985)**THETA(11)*(1+(PNA-5)*THETA(12))

CL2= TVCL2 *EXP(ETA(3)); Renal CL of paracetamol-glucuronide

FRAC1=THETA(19)

V2=FRAC1*V1

S2=V2

;Paracetamol-sulfate parameters

TVCL4=THETA(9) *(BW/985)**THETA(11)*(1+(PNA-5)*THETA(12))

CL4=TVCL4*EXP(ETA(3));Renal CL of paracetamol-sulfate

FRAC2=THETA(20)

V3=FRAC2*V1

S3=V3

;Paracetamol-oxidation parameters

TVCL6=THETA(10) *(BW/985)**THETA(11)*(1+(PNA-5)*THETA(12));

CL6=TVCL6*EXP(ETA(3));Renal CL of paracetamol-oxidative metabolites

FRAC3=THETA(21)

V4=FRAC3*V1

S4=V4

;Formation clearance

RFG=THETA(13)*EXP(ETA(4))*(BW/985)**(THETA(14));Fold difference of glucuronidation CL relative to the renal CL of unchanged PCM

RFS=THETA(15)*EXP(ETA(5))*(1-PNA/(THETA(16)+PNA) );Fold difference of sulfation CL relative to the renal CL of unchanged PCM

RFO=THETA(17)*EXP(ETA(6)) ;Fold difference of oxidation CL relative to the renal CL of unchanged PCM

RFT=RFG+RFS+RFO+1

FG=RFG/RFT; Fraction of glucuronidation relative to total PCM CL

FS=RFS/RFT; Fraction of sulfation relative to total PCM CL

FOX=RFO/RFT; Fraction of oxidation relative to total PCM CL

FR=1/RFT; Fraction of PCM renal clearance relative to total PCM CL

CL3=FG*CL1; Glucuronidation CL

CL5=FS*CL1; Sulfation CL

CL7=FOX*CL1; Oxidation CL

CLR=FR*CL1; Renal CL of unchanged PCM

K12=CL3/V1 ; Rate constant from PCM to GLUC

K13=CL5/V1 ; Rate constant from PCM to SULF

K14=CL7/V1 ; Rate constant from PCM to OXI

K15= CLR/V1 ; Rate constant from PCM to urine

K26= CL2/V2 ; Rate constant from GLUC to urine

K37=CL4/V3 ; Rate constant from SULF to urine

K48=CL6/V4 ; Rate constant from OXI to urine

K19= (Q/V1) ; Rate constant from PCM central to PCM peripheral

K91= (Q/V9) ; Rate constant from PCM peripheral to PCM central

;Urine

S5 = UVOL*THETA(18)*EXP(ETA(7))

S6 = UVOL*THETA(18)*EXP(ETA(7))

S7 = UVOL*THETA(18)*EXP(ETA(7))

S8 = UVOL*THETA(18)*EXP(ETA(7))

$ERROR (OBSERVATION ONLY)

IPRED=LOG(0.000001)

IF (F.GT.0) IPRED=LOG(F)

CT1 = 0

IF (CMT.EQ.1) CT1=1

ADD1=(1-ASY)*THETA(22)+ASY*THETA(26) ;ASY indicates dataset, 0 for dataset1 and 1 for dataset2

CT2 = 0

IF (CMT.EQ.2) CT2=1

ADD2=(1-ASY)*THETA(23)+ASY*THETA(27)

CT3 = 0

IF (CMT.EQ.3) CT3=1

ADD3=(1-ASY)*THETA(24)+ASY*THETA(28)

PRO3=(1-ASY)*THETA(31)

CT4 = 0

IF (CMT.EQ.4) CT4=1

ADD4=(1-ASY)*THETA(25)+ASY*THETA(29)

PRO4=(1-ASY)*THETA(32)

ADD=CT1*ADD1+CT2*ADD2+CT3*ADD3+CT4*ADD4

PRO=CT3*PRO3+CT4*PRO4

;Urine

CT5=0

CT6=0

CT7=0

CT8=0

IF(CMT.EQ.5) CT5=1

IF(CMT.EQ.6) CT6=1

IF(CMT.EQ.7) CT7=1

IF(CMT.EQ.8) CT8=1

ADD5=THETA(30)

ADD6=THETA(30)

ADD7=THETA(30)

ADD8=THETA(30)

ADDU=CT5*ADD5+CT6*ADD6+CT7*ADD7+CT8*ADD8

Y=IPRED+SQRT(ADD**2+ADDU**2+PRO**2/F**2)*EPS(1)

IRES=DV-IPRED

DEL=0

IF(IPRED.EQ.0)DEL=1

IWRES=(1-DEL)*IRES/(IPRED+DEL)

$DES

DADT(1)=-A(1)*(K19+K15+K12+K13+K14)+A(9)*K91

DADT(9)=A(1)*K19-A(9)*K91

DADT(2)=A(1)*K12-A(2)*K26

DADT(3)=A(1)*K13-A(3)*K37

DADT(4)=A(1)*K14-A(4)*K48

DADT(5) = A(1) * K15

DADT(6) = A(2) * K26

DADT(7) = A(3) * K37

DADT(8) = A(4) * K48

$THETA

;Fixed effect

(0, 0.139) ;PCM-CLT

(0, 0.974) ;PCM-V1

(0, 0.238) ;PCM-V2

(0, 0.0482) ;PCM-Q

(0,0.901) ;BW_PCM_CLT

(0.0458) ;PNA_PCM_CLT

(0,0.843) ;WT_PCM_V1

(0,0.0419) ;GLU-CLR

(0, 0.0264);SULF_CLR

(0,0.0517) ;OXI_CLR

(0, 1.6) ;BW_CLR

(0.0825) ; PNA_CLR

(0, 0.987) ;RFG

(0,0.22);BW_RFG

(0, 23.3) ;RFS

(0,11);PNA_RFS

(0, 3.65) ;RFOX

(0, 3.6) ;Correction Factor for urine volume

(0, 0.493) ;Fraction of VG

(0, 0.327) ;Fraction of VS

(0, 0.83) ;Fraction of VO

;Residual error

(0, 0.168) ;SD1 PCM Data1

(0, 0.31) ;SD1 GLU Data1

(0, 0.169) ;SD1 SULF Data1

(0, 0.21) ;SD1 OXI Data1

(0, 0.344) ;SD1 PCM Data2

(0, 0.843) ;SD1 GLU Data2

(0, 0.325) ;SD1 SULF Data2

(0, 0.37) ;SD1 OXI Data2

(0, 0.106) ;SD1 URINE

(0, 0.459) ;SD2 SULF Data1

(0, 0.0461) ;SD2 OXI Data1

$OMEGA

0.0908 ;IIV CL1

0.077 ;IIV V1

$OMEGA

0.141 ;IIV CLR

$OMEGA

0.29 ;IIV RFG

0.181 ;IIV RFS

0.333 ;IIV RFO

$OMEGA

1.06 ; IIV on CF

$SIGMA

1 FIX

$EST METHOD=1 INTER MAXEVAL=9999 NOABORT NSIG=2 SIGL=6 PRINT=1 POSTHOC

$COV PRINT=E MATRIX=R
